# The living experience of First Nations Peoples and Forensic Mental Health systems: listening to the deep stories behind the numbers

**DOI:** 10.1080/13218719.2024.2330476

**Published:** 2024-05-05

**Authors:** Elizabeth McEntyre, Georgia Lyons, Anina Johnson, Kimberlie Dean

**Affiliations:** a Aboriginal-Led Research Consultant, Tea Gardens (Worimi Country), NSW, Australia; b Discipline of Psychiatry & Mental Health, School of Clinical Medicine, Faculty of Medicine and Health, University of New South Wales, Kensington, NSW, Australia; c Justice Health and Forensic Mental Health Network, Sydney, NSW, Australia

**Keywords:** First Nations People, forensic mental health, forensic patient, lived experience, psychiatry

## Abstract

While First Nations Peoples in Australia experience high rates of criminal justice contact, there is limited research on their experiences of the forensic mental health system. This study aims to develop new understandings of how First Nations Peoples experience and understand the forensic mental health system in NSW. Interviews were conducted with ten First Nations Peoples in contact with the forensic mental health system, including forensic patients and their family members. Participants described challenging life experiences prior to their contact with the forensic mental health system, with community services often failing to respond to their mental health needs. While participants reported some positive experiences with the forensic mental health system, they ultimately described an urgent need for culturally appropriate programs that facilitate connections to family and Community. Forensic mental health services should be co-designed alongside First Nations Peoples and communities to improve outcomes and avoid re-traumatisation through contact with services.

## Introduction

Mental illness is known to be associated with an elevated risk of contact with criminal justice systems (Fazel et al., 2009; Yee et al., 2020). For less serious crimes, many jurisdictions facilitate diversion from courts and prison for those with severe mental illness on the basis that appropriate care and treatment is required rather than custodial punishment or other sanction. For people with severe mental illness who are found to have committed serious crimes, most common law jurisdictions allow a court to find that a person is not criminally responsible because their mental illness prevented their understanding of the nature of the conduct. In New South Wales (NSW), the verdict was known as not guilty by reason of mental illness (NGMI), and those who receive this verdict are referred to as ‘forensic patients’.^1^ In NSW, the Mental Health Review Tribunal (MHRT) is responsible for reviewing the care, treatment and detention of these forensic patients. Most NSW forensic patients will receive mental health care in a forensic mental health facility. However, bed availability means that forensic patients will also generally spend several years in prison after the index offence and before moving to a forensic mental health facility (NSW Mental Health Review Tribunal (NSW MHRT), 2022).

First Nations Peoples in Australia (i.e., Aboriginal and/or Torres Strait Islander Peoples) are overrepresented within the criminal justice system, including in their rates of contact with police, courts, and prisons (AIHW 2020; Justice Health & Forensic Mental Health Network, 2015; McCausland et al., 2017). The high rates of mental illness among First Nations men and women in the community are also well established, with the Australian Institute of Health and Welfare (AIHW) estimating that 24% of First Nations Peoples have a mental health or behavioural condition (Australian Institute of Health and Welfare (AIHW), 2020). In addition to the high rates of mental illness experience by First Nations Peoples in contact with the criminal justice system, elevated rates of physical health problems and social disadvantage are also apparent (Baldry et al., 2015; McCausland et al., 2017).

The overrepresentation of First Nations Peoples with mental illness within the criminal justice system must be understood within the context of ongoing colonisation and the continued practices of racism and dispossession faced by First Nations Peoples today (Australian Law Reform Commission (ALRC), 2018). The impacts of colonisation, including the dispossession of land and waters, disruption of culture and family systems, removal of children and institutionalisation, has made and continues to make a strong contribution to the elevated rates of contact with the criminal justice system for First Nations Peoples (Aboriginal Justice Victoria, 2022).

Despite their overrepresentation within the criminal justice system, First Nations Peoples appear to be comparatively underrepresented within the forensic mental health system. In 2016, the NSW Justice Health and Forensic Mental Health Network estimated that 16.8% of forensic patients in high secure settings identified as Aboriginal and/or Torres Strait Islander (Justice Health & Forensic Mental Health Network, 2016). This is significantly lower than the proportion of First Nations Peoples in prison, which the Australian Bureau of Statistics (ABS) estimates to be 29% (Australian Bureau of Statistics (ABS), 2020). In another study examining the predictors of mental health diversion among a cohort of offenders with mental illness presenting to Local Court with lesser charges, Aboriginal and/or Torres Strait Islander people were found to be less likely to receive a diversion (Soon et al., 2018).

The impacts of colonisation, discrimination and inadequate cultural sensitivity and cultural responsiveness within the criminal justice and forensic mental health systems may exacerbate the impact of mental illness among First Nations Peoples (Durey et al., 2015). Previous negative experiences and distrust of services may also impact First Nations Peoples’ engagement with the health system (Jones et al., 2020). In a survey conducted by the NSW Justice Health and Forensic Mental Health Network, 53.3% of Aboriginal participants reported experiencing racial discrimination in the 12 months preceding the survey, including being ignored or treated with suspicion (Justice Health & Forensic Mental Health Network, 2016). In another study exploring the experiences of First Nations Peoples with mental illness and cognitive disability in contact with the criminal justice system, institutional racism was experienced as pervasive for Aboriginal people in contact with services (Baldry et al., 2015). This included services having a lack of understanding of the intergenerational impact of trauma, grief and loss, and the lack of funding and support for culturally appropriate services. Participants in the study also discussed the way in which the lives of First Nations People in contact with these systems were often marked by poverty, instability and violence, with the absence of appropriate early diagnosis and positive culturally responsive support. Health professionals working in the forensic mental health system have also reported challenges in delivering high quality care for First Nations patients, including a lack of resources to meet individual patient needs, inadequate staffing levels and the need to better connect with Aboriginal health and support services (Durey et al., 2015).

While there is some emerging evidence of the experiences of First Nations Peoples within the forensic mental health system in Australia, qualitative research that explores the lived experience of First Nations Peoples enmeshed with this system is extremely limited. A previous quantitative study, utilizing data from the NSW Forensic Patient Database, identified a profile of complex needs and poorer post-release outcomes for First Nations forensic patients compared to non-First Nations patients (Dean et al., 2023). Beyond such findings, a deeper understanding of the lived experiences and stories of First Nations Peoples is crucial for the development of culturally informed policies, practices and standards that can be applied to achieve better outcomes. The current study aimed to develop new understanding of how First Nations Peoples experience and understand the forensic mental health system in NSW, through a qualitative investigation in partnership with those who have been directly involved with this system as forensic patients and their family members.

## Methods

### First Nations Community and cultural governance arrangements

The First Nations Community and cultural governance arrangements for the project were described in detail in Dean et al. (2023). In summary, the research was initiated and co-led by the lead author (EM), who is a Worimi and Wonnarua Woman and Elder, alongside the academic lead for the NSW Forensic Patient Database Project (KD). The project reflected current and unrelenting concerns from First Nations Communities in NSW regarding their experiences of the criminal justice and forensic mental health systems (McEntyre, 2019). EM engaged and consulted with Mindaribba Local Aboriginal Land Council (located on Wonnarua Country) and Biripi Aboriginal Corporation Medical Centre (ACMC) (located on Biripi Country) who provided formal support for the project. Specifically, the project was supported by Biripi ACMC and endorsed and overseen by the Board of Mindaribba Local Aboriginal Land Council. Mindaribba has overall sovereign rights and control of the data, and the data is securely stored at the University of New South Wales (UNSW).

All aspects of the study were designed in consultation with First Nations organisations and community members who provided input on the research questions, data analysis, interpretation of the results, and publication of the findings. The project adhered to all ethical requirements outlined by the Aboriginal Health and Medical Research Council (AHMRC) Human Research Ethics Committee (HREC).

### Recruitment

Qualitative interviews were conducted to explore First Nations Peoples’ experiences of the forensic mental health system in NSW. Participants were eligible for inclusion in the study if they were aged at least 18 years old or older and identified as First Nations and were either currently being treated in secure care after an NGMI finding or were a family member of someone being treated in secure care.

Participants were recruited from a large Australian-based secure psychiatric hospital providing treatment for forensic patients. Participant recruitment involved a collaborative approach with hospital staff and First Nations forensic patients. A First Nations researcher attended the hospital to meet with an Aboriginal Mental Health Care Worker and other relevant practitioners to discuss the aims of the project. Following this, the researcher engaged with the First Nations forensic patients during their weekly ‘Yarning’ group session to explain the aims of the research and invite them to participate in an interview at a later date. Advice was then sought from the Aboriginal Mental Health Care worker to identify any potential issues relating to capacity, risk, and distress for potential participants. Participants were provided with an easy-read Participant Information Statement and Consent form that outlined the aims and requirements of the study.

### Data collection

A total of 10 individuals participated in an interview. All interviews were conducted by the first author (EM), who is a highly experienced First Nations researcher and Elder. All eligible First Nations patients at the hospital agreed to participate in an interview, with the exception of one who was thought to be not well enough to participate at the time. Face-to-face interviews were conducted with eight forensic patient participants in the presence of the Aboriginal Mental Health Care Worker. Interviews with two family members were conducted face-to-face or by telephone. Interviews were conducted using a semi-structured interview schedule to explore the forensic patients’ experiences with the criminal justice system and forensic mental health system, their broader life course experiences and associated support needs, the types of services received throughout their life, their experiences of these services and their thoughts on the type of services they needed and those that would have been helpful at key times. A comparable approach was taken to the qualitative interviews with family members who were first identified by the First Nations Forensic Patients. All interviews were digitally recorded and transcribed by a professional transcription agency.

The research team ensured that the data collection process was culturally safe for the participants. The strong and trusting relationship between the participants and the Aboriginal Mental Health Care Worker, together with the first author’s cultural and systemic knowledge and professional and personal experience, ensured that participants did not feel shame or confront stigma when sharing their life, story and truth. The participants showed no concern when speaking about their lives, as this was the first genuine opportunity they had been given to speak openly and directly to an Aboriginal person who was not connected to the forensic mental health system like they were who, however, knew the system well.

### Analysis

Thematic analysis was used to identify key issues discussed by the interview participants and to identify emerging patterns, variability and consistency, commonality and differences between the participants (Braun & Clarke, 2006). The data were analysed inductively without a pre-determined coding frame to ensure that the findings were strongly driven by the experiences of the participants. After becoming familiarised with the data, the codes and themes were generated and reviewed and agreed upon by the authors (EM, GL). The interview data was analysed using NVivo 12 software (QSR International, 2018).

## Results

Table 1 outlines the themes captured from the interviews with First Nations forensic patients and their family members. Interviews provided insight into the journey of First Nations forensic patients that had led them to the forensic mental health system. The distinct stages of these journeys included: (1) life before entering the prison system, (2) life in the prison system, (3) experiences of the forensic mental health system, and, (4) working towards living in the community. Key themes are illustrated below using direct interview quotes from the participants.

**Table 1.** Key themes developed from the interview data.

Stage in the participants’ journey	Key themes
Life before entering the prison system	Difficulties with learning at school Experiences of violence Experiences of poverty Exposure to alcohol and drugs Experiences of kinship care Aspirations for the future Lack of support Importance of sporting or cultural activities
Life in the prison system	Psychological impacts, including the exacerbation of pre-existing mental health issues Cycling in and out of prison Lack of support in addressing mental health issues Lost contact with family members
Experiences of the forensic mental health system	Ongoing cycle of attending Tribunal hearings without feeling any sense of progress Regretting pleading NGMI Lack of understanding about the consequences of an NGMI verdict Importance of having Aboriginal Mental Health Care Workers More cultural programs and guidance from Community Elders is needed Hospital programs and activities become repetitive and unhelpful Aspiring to improve skills, including reading and writing Difficulty relating to health professionals Lack of communication with family members
Working towards living in the community	Importance of supportive housing and services Importance of effective medication regime Reconnecting with family and Community

### Life before entering the prison system

Participants were asked to discuss their early life experiences, including their relationship with their family, schooling experiences, exposure to the criminal justice system, and whether they received support from any services. Their responses provided insight into the intergenerational trauma that is likely to have contributed to their contact with the criminal justice system and forensic mental health systems later in life. Participants experienced a range of challenges during their childhood. This included difficulties with learning at school, poverty, violence, sexual assault, exposure to alcohol and drug use, and being placed in kinship care. Several participants described how their disengagement from school and exposure to violence contributed to their trajectory into the criminal justice system:

At the age of 14, I started skipping school more … I was doing more drugs, I was doing crime … I was sexually assaulted at the age of 14 and sexually assaulted at the age of 17.

I didn’t do good at school. I had ADHD so I wasn’t very good at listening. I got into a lot of trouble at school. I got expelled … I’d get in trouble [with the police] at the age of six or seven. They couldn’t charge me, so they talked to my nan and my nan had custody of me … I couldn’t read or write at all.

At the same time, participants had desires and aspirations for their life before coming into contact with the criminal justice system:

I would’ve liked to become a welfare worker for the young Aboriginal kids. That would’ve been good. I didn’t have the education, so I couldn’t really do it. It’d be good to do something like that.

When I was a kid, I’d wished that I was a police officer, stop all the Aboriginal kids getting locked up and that, look after them when they go to the police cells … that’s what I really loved to be when I was a kid.

Participants were asked to consider the types of mental health support they had received from services during their childhood years. There was a consensus that mental health services were either not available or were not helpful. However, some participants spoke positively of the cultural activities that were available during their childhood. This included Aboriginal camps and learning culture through dance. One participant described their eventual contact with the criminal justice system once these activities were discontinued:

They had opened up an Aboriginal thing for young kids, to learn about Aboriginal dance, about our culture. I spent most of my time at Aboriginal camps, travelling around Australia, dancing and stuff like that. I loved it … Ended up getting closed down, so I ended up getting knocked into juvie.

Other participants described the importance of their family in supporting them during their childhood:

My foster family were very, very special to me. They used to help me a lot … They put me in school. They fed me. They loved me. They clothed me. They let me go out places.

Participants were asked to consider the types of support they wished they had received during their childhood. Examples identified by the participants included drug and alcohol support programs and opportunities for children to be engaged in the community, such as in sporting or cultural activities. Support services for families were also identified as crucial in addressing the impacts of intergenerational trauma. As one family member of a forensic patient described, families may be in crisis and require immediate support:

Everyone’s got trauma. Continually handed down. … There’s always got to be some sort of service or someone there for these families to help guide them. … It’s got to be ongoing. … I can’t figure out with psychologists or psychiatrists when I take kids to their appointments. [The kids] need to see [the psychologist] again tomorrow or the next day, not in another month. … Too many crises happen in-between.

### Life in the prison system

Nearly all the forensic patients who participated in the study had been involved in the criminal justice system before being diverted into the forensic mental health system. Participants who did not have contact with the criminal justice system as young people had memories of family and community members being involved with police and prison. Participants’ experiences of prison had a significant impact on their psychological wellbeing, with many describing their first experience of prison as frightening. One participant described their first experience of prison as follows:

When I first came in, they gave me a cell and it was too small for me. And I’d keep hitting the door and say ‘let me out, let me out, let me out. I’m dying in here. It’s too small. It’s too small. … the cell they put me in, I could have died.

One participant experienced segregation while in prison, which had considerable negative impacts for their mental health:

[Segregation in prison] was very, very bad. … Four walls, nothing to watch … it was just so bad … there was just cameras, toilet, a bed, no exercising yard, nothing at all for two whole years. Then we got a TV in like a cabinet thing … and then the courtyard out the back. But that took two and a half years just to get.

Participants often cycled in and out of prison over several years without receiving the mental health support that they needed. It was often known that the person had a mental illness but they were not given support to attend medical appointments and maintain an effective medication regime. This was illustrated by the experience of one participant, who at first experienced very brief periods of imprisonment but the periods became more prolonged over time:

First five times [in prison] I was only in for about a few days to a week … sixth time it was three years, and the seventh time I went in for years, a long time … they weren’t feeding me, they weren’t clothing me. … I wasn’t getting stuff to shower or fresh clothes. That’s when I went crazy.

### Experiences of the forensic mental health system

Participants had mixed opinions about the benefits of being in the forensic mental health system compared to the criminal justice system. While they were in prison, some participants thought that being in the forensic mental health system would be better, as they would be able to participate in more activities and access day leave. One participant had spent several years in prison before being transferred to a secure mental health facility and reflected that the forensic mental health system was ‘not what it was made out to be’. Another participant discussed how she was essentially lost in the system once she went to hospital:

My sister and that helped me, but then they lost contact with me because they didn’t know where I was. When I moved [to hospital], they thought I was still in jail. So they’ll ring the jail to find out where I was. They said I’m not in the system no more, they don’t know where I am.

Several participants regretted pleading NGMI, as upon reflection they would have preferred to serve a prison sentence rather than being ‘stuck’ or held indefinitely in a secure mental health facility. Some participants stated that they had not understood the implications of an NGMI verdict at the time. Participants were also frustrated by the ongoing cycle of attending MHRT hearings every six months without any sense of progress. One participant described the lack of support from people involved in the Tribunal hearings:

No one’s out there for us, to help us. The Tribunal says another six months, another six months. And I’m still here, waiting for them to let me out. … I’ve done all the programs, what they want me to do. I’ve done everything they want me to do.

Despite this, some participants did report positive experiences of the secure mental health facility in which they were held. One participant appreciated that they were prescribed the correct medication when they arrived at the hospital, which meant that they could manage the symptoms of their mental illness more effectively. However, some participants reported that their experience of hospital was difficult due to the negative side effects of medication and the threat of being placed in isolation.

Participants valued having an Aboriginal Mental Health Care Worker who would advocate for the patient’s rights and wellbeing, organise cultural activities and liaise with the patient’s families:

The Aboriginal Mental Health Care Worker has a good position. They talk to us when they can and have a good rapport with us. And they helped me to paint for Reconciliation Week.

However, participants reported that there was often a high turnover of Aboriginal staff which made it difficult to build rapport. Participants suggested that the high turnover was likely due to the pressures of the job and the challenges of being the only Aboriginal staff member in the team:

[The Aboriginal Mental Health Care Worker] didn’t last long. They’d come in, they were here for a week, they didn’t like how we were being treated or how they got spoken to … because they try and speak up for us and get shut down by the nurses or managers.

Participants had mixed views on the activities and programs offered at the hospital, which included fitness groups, art classes and skills-building programs. However, one participant noted that more cultural programs were needed:

More cultural programs would be good … art programs or something to do with the Dreaming time stories … more language, Elders visiting too, music and dance, [I would] like to learn some songs.

Participants reported a desire to improve their skills while they were at the hospital, including reading and writing skills. However, as one participant noted, there appeared to be limited resources for these activities:

There is no teacher to [help us learn to] read and write. I said to the doctors … ‘I’m pretty smart with numbers and that … I want to learn to read and write’. They said, ‘we’ll see about it, we’ll see about it’. And they’re still seeing about it, and it’s been like three or four years now.

Participants also reported difficulties in engaging with the health professionals at the hospital, particularly the non-Aboriginal workers. Participants felt that many of the doctors and nurses did not have a proper understanding of their health and wellbeing needs. One participant stated that they only met with their psychiatrist ‘once in a blue moon’. Several participants gave specific examples of negative experiences with health professionals:

[The nurses] just don’t listen to us … you ask for support and they don’t want to give it to us. They want to brush us off, leave us the way we are.

The doctor just keeps pushing me [into other units] every time I see her. It’s changing all the time … she says one thing and does a different thing.

While family member participants were relieved to know that the patient was in a safe place, they thought that the communication between the hospital and families could be improved. Family members often had no understanding of what was happening to the forensic patient. Having no understanding of the term ‘forensic’ and the location or operations of the hospital often resulted in feelings of confusion and worry. One participant reported challenges in organising visits at the hospital, particularly when young children were involved, due to limits on the number of people who could visit the patient at one time and the daunting security screening process for visitors. Family members also thought that Aboriginal workers were essential, as they were able to facilitate connections between patients, their families and health professionals.

### Working towards living in the community

During the interviews, forensic patient participants expressed their desire to leave the hospital and return to living in the community. Supportive housing, support services and an appropriate medication regime were considered crucial for their transition to the community. Some participants described how they were working with the National Disability Insurance Scheme (NDIS) to access supported independent housing in the community:

NDIS are working with me to try and get me out of here. I’ve nearly got my house ready as far as I know. And they said they just want a good life for me because I’m getting older.

Many participants had lost contact with family at some point during their journey. Some participants expressed their desire to eventually reconnect with their family once they were released from hospital, highlighting the importance of family and kinship connections for forensic patients upon return to the community:

You know there should be places that you go when you leave these places and stuff. But there’s also things like being in touch with your family. That’s the most important thing … that’s why I want to leave this place.

Participants were asked to reflect upon the types of support that First Nations Peoples need when living in the community, to prevent them from re-entering the criminal justice and forensic mental health systems. Timely access to culturally safe and responsive wrap-around services were considered essential for addressing the complex support needs of First Nations forensic patients. This includes services that address alcohol and other drug issues and intergenerational trauma, as these can exacerbate symptoms of mental illness:

We need to show the world and the system that by helping Aboriginal people, from wherever they are in the community or whatever community they’re in, they’ve got to be taught the right way and we’ve got to be shown that drugs and alcohol are a big major factor and we don’t want to play a part in that. They bring it to us, the white fellas.

Participants also discussed the importance of fostering connections between Elders and young First Nations Peoples, to allow them to reconnect to their culture:

Teach kids about culture … they need to listen … the activities side and the positive side of it as well … we went through the massacres and all that. And then through the surviving and that … it’s a dark past but there’s a bright future.

## Discussion

This qualitative study of the experiences and views of First Nations forensic patients and their family members is the first such investigation to be conducted in Australia. A range of key themes emerged across identified life stages, from life before entering prison, through prison and the forensic mental health system, and plans for returning to Community. Study participants reported life stories that contained multiple layers of hurt and hardship from an early age combined with limited or no contact with services that were properly equipped to respond to their complex support needs. Participants reported mixed experiences with the forensic mental health system itself. While participants valued having an Aboriginal health care worker who advocated for them, participants also reported negative interactions with other health professionals and felt that these professionals did not understand their needs. Participants expressed a desire to leave the hospital and return to their Community and re-connect with family. The lived experiences of participants highlighted the importance of co-designing forensic mental health and other services alongside First Nations Peoples and communities in order to improve outcomes and avoid re-traumatisation through contact with these systems.

### Main findings

The themes emerging with regard to experiences of hardship in early life, before contact with the criminal justice system, are consistent with previous research examining the life-course experiences of other First Nations Peoples with mental illness in contact with the criminal justice system (Baldry et al., 2015). It is also consistent with the quantitative study findings from the NSW Forensic Patient Database study linked to the current study, which found that First Nations forensic patients were more likely to have experienced sociodemographic disadvantage and past trauma compared to non-First Nations forensic patients (Dean et al., 2023). The consequences of such early life hardship, including the resulting unmet support needs, are likely to have contributed to the risk of contact with the criminal justice system and to the index offence itself. All forensic patient participants had experienced imprisonment before being diverted to the forensic mental health system, often cycling in and out of prison and losing contact with family members and community. These experiences are likely to have exacerbated pre-existing mental health issues that were not properly diagnosed or treated while in prison, highlighting the systemic lack of culturally safe support for First Nations Peoples with a mental illness. Several participants expressed their regret about pleading NGMI which ultimately led to their indefinite detention in secure care. These findings suggest that First Nations Peoples and their families and advocates may not be sufficiently informed about the forensic mental health system and the implications of receiving an NGMI defence.

Participants reported both positive and negative experiences from their time at the secure psychiatric hospital. Some participants described the programs and activities offered at the hospital as helpful, while others felt that they were repetitive and were not meeting their needs. This points to the need to develop and deliver services for First Nations forensic patients that are culturally informed and competent, empathic, and adopt a holistic approach to health and wellbeing (Kendall & Barnett, 2015). Participants in the current study made a range of suggestions such as a purpose-built sacred space to support individual and collective healing, strengths and solution-focused ‘yarn ups’, and celebrations for significant cultural events. In order for such programs to be effective, they should address issues identified by local communities, be driven by local leadership, be understanding of the impact of colonisation and intergenerational trauma and build upon individual, family and community capacity (McKendrick et al., 2013). Participants also expressed a strong desire for more engagement with Community Elders, who could share their cultural knowledge and guidance to support First Nations forensic patients during their time in the secure hospital, providing a link to Community and helping them to prepare for their return to the community. Existing evidence from Australia and internationally demonstrates the effectiveness of partnering with First Nations Elders to deliver mental health care in improving outcomes for service users (Hadjipavlou et al., 2018; Tu et al., 2019) and assisting non-First Nations staff to improve their understanding of First Nations culture, kinship and importance of Country and Community to Aboriginal wellbeing (Wright et al., 2021).

While participants valued having an Aboriginal Mental Health Care Worker who could advocate on their behalf, participants reported an insufficient number of such staff and significant challenges engaging with non-Aboriginal staff. Previous research indicates that improving forensic mental health care for First Nations Peoples, non-Aboriginal workers require more than one-off cultural competence training; they need, rather, ongoing education and support in order to provide culturally safe mental health care, (Durey et al., 2015). In addition, it is vital that First Nations staff are not isolated within services and that their knowledge, skills and experiences are leveraged to build culturally safe and responsive practices among their non-Aboriginal colleagues.

The study also provides some insight into the apparent underrepresentation of First Nations Peoples with the forensic mental health system compared to the criminal justice system. The findings indicate that some First Nations Peoples may prefer, or are advised by legal professionals, to serve a sentence of imprisonment rather than seek a mental health defence/disposal, which entails an indefinite period of detention in secure care. It is also possible that First Nations Peoples in prison are more likely to be charged with less serious offences (ALRC, 2018) that do not qualify for an NGMI defence. This issue ultimately warrants further investigation to ensure that First Nations Peoples with mental illness receive appropriate receive care and support.

While this study highlights the complex and pervasive experiences of hardship and disadvantage among First Nations forensic patients, participants drew upon a range of strengths throughout their journeys in the criminal justice and forensic mental health systems that should not be overlooked. Participants discussed the value of cultural activities that they were involved with during their childhood, such as art and dance, which enabled them to connect with land and country. Many participants discussed aspirations to work with their Community and improve the lives of First Nations Peoples. Adopting a culturally informed and strengths-based approach to forensic mental health care, which highlights the knowledge, resources and agency of First Nations Peoples, is critical to improving outcomes both in secure care and upon release to the community.

### Strengths and limitations

The current study benefited from several strengths. It is the first study conducted in NSW that comprehensively examines the lived experiences of First Nations forensic patients who are detained in secure care, as well as their family members. By adopting a life-stage approach to the interviews, the study has identified several key recommendations that may be adopted to improve outcomes for First Nations People. The study was co-designed and governed by First Nations Peoples and communities who provided expert advice and consultation throughout the course of the project.

The limitations of the present study must also be noted. The study utilised a relatively small sample size of 10 participants. While the study findings were consistent with previous research examining First Nations Peoples experiences of the criminal justice system more broadly, the recruitment of a larger sample size may have allowed the researchers to identify additional themes and trends in the data. The extent to which the findings of the present study can be generalised to First Nations populations internationally is also limited, given that legal and service approaches to forensic patients vary widely across jurisdictions.

## Conclusion

The findings of this study confirm the complex and culturally specific support needs of First Nations forensic patients that require specific responses informed by the well-known historical, physical, social, economic and cultural determinants of health and wellbeing for First Nations communities. Participants described challenging journeys leading up to the index offence, marked by poverty, violence, exposure to drug and alcohol use, early contact with the criminal justice system, and the ultimate failure of community services to adequately respond to their mental health needs. This study also begins to address a significant gap in the evidence base regarding the experiences of First Nations Peoples within the forensic mental health system, by highlighting the need for more culturally appropriate programs led by First Nations staff and that involve strong connections to Community. Providing holistic care and services that are trauma-aware and healing-informed and have a strong focus on the social and emotional wellbeing of the person and their family, is essential for people in the forensic mental health system and those receiving mental health services more generally. First Nations forensic patient outcomes will only be improved if the care and treatment they receive takes findings like these into account: care and treatment that is timely, culturally safe and responsive, and informed by lived experience.

## Footnotes

1 In March 2021, the finding of ‘NGMI’ was replaced with the finding of ‘act proven but not criminally responsible’ under the *Mental Health and Cognitive Impairment Forensic Provisions Act 2020* (NSW). The authors have chosen to retain the term ‘NGMI’ in this article as all study participants received an NGMI verdict under the original legislation.
